# Patterns in COVID-19 Vaccination Coverage, by Social Vulnerability and Urbanicity — United States, December 14, 2020–May 1, 2021

**DOI:** 10.15585/mmwr.mm7022e1

**Published:** 2021-06-04

**Authors:** Vaughn Barry, Sharoda Dasgupta, Daniel L. Weller, Jennifer L. Kriss, Betsy L. Cadwell, Charles Rose, Cassandra Pingali, Trieste Musial, J. Danielle Sharpe, Stephen A. Flores, Kurt J. Greenlund, Anita Patel, Andrea Stewart, Judith R. Qualters, LaTreace Harris, Kamil E. Barbour, Carla L. Black

**Affiliations:** ^1^Epidemic Intelligence Service, CDC; ^2^CDC COVID-19 Response Team; ^3^Geospatial Research, Analysis, and Services Program, Agency for Toxic Substances and Disease Registry, Atlanta, Georgia; ^4^Division of Population Health, National Center for Chronic Disease Prevention and Health Promotion, CDC.

Disparities in vaccination coverage by social vulnerability, defined as social and structural factors associated with adverse health outcomes, were noted during the first 2.5 months of the U.S. COVID-19 vaccination campaign, which began during mid-December 2020 (*1*). As vaccine eligibility and availability continue to expand, assuring equitable coverage for disproportionately affected communities remains a priority. CDC examined COVID-19 vaccine administration and 2018 CDC social vulnerability index (SVI) data to ascertain whether inequities in COVID-19 vaccination coverage with respect to county-level SVI have persisted, overall and by urbanicity. Vaccination coverage was defined as the number of persons aged ≥18 years (adults) who had received ≥1 dose of any Food and Drug Administration (FDA)-authorized COVID-19 vaccine divided by the total adult population in a specified SVI category.^†^ SVI was examined overall and by its four themes (socioeconomic status, household composition and disability, racial/ethnic minority status and language, and housing type and transportation). Counties were categorized into SVI quartiles, in which quartile 1 (Q1) represented the lowest level of vulnerability and quartile 4 (Q4), the highest. Trends in vaccination coverage were assessed by SVI quartile and urbanicity, which was categorized as large central metropolitan, large fringe metropolitan (areas surrounding large cities, e.g., suburban), medium and small metropolitan, and nonmetropolitan counties.^§^ During December 14, 2020–May 1, 2021, disparities in vaccination coverage by SVI increased, especially in large fringe metropolitan (e.g., suburban) and nonmetropolitan counties. By May 1, 2021, vaccination coverage was lower among adults living in counties with the highest overall SVI; differences were most pronounced in large fringe metropolitan (Q4 coverage = 45.0% versus Q1 coverage = 61.7%) and nonmetropolitan (Q4 = 40.6% versus Q1 = 52.9%) counties. Vaccination coverage disparities were largest for two SVI themes: socioeconomic status (Q4 = 44.3% versus Q1 = 61.0%) and household composition and disability (Q4 = 42.0% versus Q1 = 60.1%). Outreach efforts, including expanding public health messaging tailored to local populations and increasing vaccination access, could help increase vaccination coverage in high-SVI counties.

COVID-19 vaccination data are reported to CDC through state, local, and territorial immunization information systems, the Vaccine Administration Management System, or direct data submission to the CDC Data Clearinghouse.^¶^ County-level data on FDA-authorized COVID-19 vaccines administered during December 14, 2020–May 1, 2021, and reported through May 5, 2021, were analyzed. County-level SVI data were obtained from the 2018 CDC SVI, which is used to prioritize public health resources for communities with the greatest needs during and following emergencies (*2*,*3*). Ranked scores ranging from 0–1 were created for all 3,142 U.S. counties based on 15 population-based social determinants of health measures, categorized into one of four themes: socioeconomic status, household composition and disability, racial/ethnic minority status and language, and housing type and transportation.** Scores for overall SVI and themes were analyzed as quartiles. The 15 individual SVI components were dichotomized at the median, based on distribution among all U.S. counties. County urbanicity was categorized as large central metropolitan, large fringe metropolitan, medium and small metropolitan, and nonmetropolitan.

Data from adults living in 3,129 (99%) U.S. counties were analyzed; California counties with populations <20,000 and all Hawaii counties were excluded because of lack of available county-level vaccination data. Vaccine recipients were categorized by SVI metrics and urbanicity, based on county of residence. Trends in vaccination coverage were evaluated by epidemiologic week for SVI quartile, stratified by urbanicity. Generalized estimating equation models using binomial regression and an identity link were used to estimate vaccination coverage by SVI metrics, overall and by urbanicity.^††^ Absolute coverage differences with corresponding 95% confidence intervals (CIs) were calculated to evaluate differences between groups. Differences in coverage by SVI were also evaluated for three separate periods to assess variation in inequities over time.^§§^ All analyses were conducted using SAS (version 9.4; SAS Institute). This activity was reviewed by CDC and was conducted consistent with applicable federal law and CDC policy.^¶¶^

During December 14, 2020–May 1, 2021, 54% of adults living in the 3,129 assessed U.S. counties received ≥1 dose of COVID-19 vaccine. Disparities in vaccination coverage by SVI increased over time, especially in large fringe metropolitan and nonmetropolitan counties, where coverage differences between SVI Q4 and Q1 counties were most prominent (Figure) (Supplementary Table, https://stacks.cdc.gov/view/cdc/106461).

**FIGURE:**
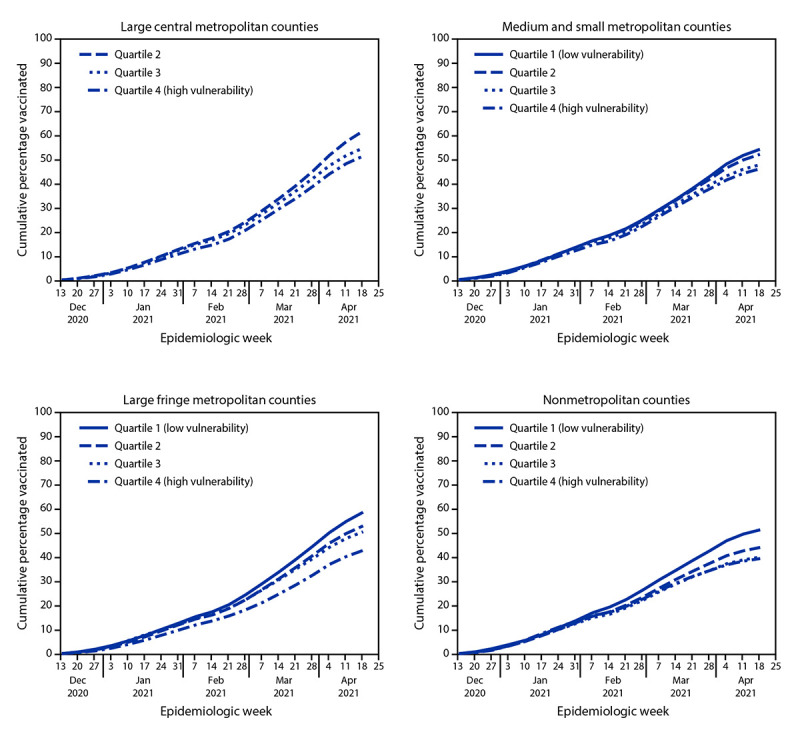
COVID-19 vaccination coverage among U.S. adults, by county social vulnerability index quartile* and urbanicity^†^ (N = 3,129 counties^§^) — United States, December 14, 2020–May 1, 2021^¶^^,^** **Abbreviation:** SVI = social vulnerability index. * Scores for all SVI measures represented percentile rankings by county, ranging from 0–1, with higher scores indicating higher vulnerability. Scores were categorized into quartiles based on distribution among all 3,142 U.S. counties and then applied to the 3,129 assessed counties. ^†^ Urbanicity categories were based on the 2013 National Center for Health Statistics urban-rural classification scheme (https://www.cdc.gov/nchs/data/series/sr_02/ sr02_166.pdf). Categories were collapsed into large metropolitan, large fringe metropolitan, medium and small metropolitan, and nonmetropolitan (micropolitan and noncore) counties. ^§^ California counties with populations <20,000 (n = 8) and all Hawaii counties (n = 5) were excluded because of lack of available county-level vaccination data. ^¶^ Only 6 days of data were available for week December 13, 2020 (analysis used data from December 14, 2020, and on). ** Results were suppressed for SVI and urbanicity categories with four or fewer counties (quartile 1, large central metropolitan counties).

By May 1, 2021, after states opened eligibility to all adults, vaccination coverage was lower among adults living in counties with the highest overall SVI (Q4 coverage = 49.0% versus Q1 coverage = 59.3%) (Table 1). Coverage differences between adults living in counties with the highest versus lowest SVI were –11.0% (95% CI = –13.2% to –8.9%) in large central metropolitan counties, –16.7% (95% CI = –20.7% to –12.7%) in large fringe metropolitan counties, –8.2% (95% CI = –13.1% to –3.4%) in medium and small metropolitan counties, and –12.3% (95% CI = –16.4% to –8.2%) in nonmetropolitan counties. Coverage differed by three SVI themes: coverage was lower in counties with higher SVI pertaining to socioeconomic status (Q4 = 44.3% versus Q1 = 61.0%) and household composition and disability (Q4 = 42.0% versus Q1 = 60.1%), but higher in counties with higher SVI related to racial and ethnic minority residents and English proficiency (Q4 = 56.5% versus Q1 = 45.3%).

**TABLE 1:** Associations between social vulnerability index* and vaccination coverage^†^ among U.S. adults, overall and by county urbanicity^§^ (N = 3,129 counties^¶^) — United States, December 14, 2020–May 1, 2021

SVI quartile	All counties		Large central metropolitan		Large fringe metropolitan		Medium and small metropolitan		Nonmetropolitan	
	(N = 3,129)		(n = 68)		(n = 368)		(n = 727)		(n = 1,966)	
	VC estimate	VC differences (95% CI)	VC estimate	VC differences (95% CI)	VC estimate	VC differences (95% CI)	VC estimate	VC differences (95% CI)	VC estimate	VC differences (95% CI)
**Overall SVI**										
Q1 (lowest)	**59.3**	**Ref**	—**	—	61.7	Ref	56.2	Ref	52.9	Ref
Q2	**56.0**	**−3.2 (−7.2 to 0.8)**	65.1	Ref	55.6	−6.1 (−10.2 to −2.0)	54.1	−2.1 (−5.9 to 1.7)	45.4	−7.5 (−10.6 to −4.5)
Q3	**52.5**	**−6.8 (−10.3 to −3.3)**	57.4	−7.7 (−11.9 to −3.5)	53.1	−8.6 (−11.7 to −5.5)	49.5	−6.7 (−10.5 to −2.9)	41.3	−11.6 (−15.4 to −7.9)
Q4 (highest)	**49.0**	**−10.3 (−14.1 to −6.4)**	54.0	−11.0 (−13.2 to −8.9)	45.0	−16.7 (−20.7 to −12.7)	47.9	−8.2 (−13.1 to −3.4)	40.6	−12.3 (−16.4 to −8.2)
**SVI related to socioeconomic status**										
Q1 (lowest)	**61.0**	**Ref**	—	—	62.2	Ref	57.1	Ref	54.7	Ref
Q2	**54.2**	**−6.8 (−9.6 to −4.0)**	59.2	Ref	51.7	−10.5 (−13.5 to −7.4)	52.9	−4.2 (−7.0 to −1.5)	46.8	−7.9 (−11.1 to −4.6)
Q3	**50.0**	**−11.0 (−13.4 to −8.6)**	55.2	−4.0 (−10.6 to 2.6)	45.0	−17.1 (−21.0 to −13.3)	46.4	−10.7 (−14.1 to −7.4)	40.9	−13.8 (−17.7 to −9.9)
Q4 (highest)	**44.3**	**−16.7 (−20.9 to −12.5)**	50.8	−8.5 (−17.3 to 0.4)	41.4	−20.8 (−26.9 to −14.6)	48.4	−8.7 (−16.2 to −1.1)	39.2	−15.5 (−19.7 to −11.3)
**SVI related to household composition and disability**										
Q1 (lowest)	**60.1**	**Ref**	—	—	61.5	Ref	56.5	Ref	50.0	Ref
Q2	**50.1**	**−10.0 (−12.6 to −7.3)**	51.7	Ref	48.6	−12.8 (−15.7 to −10.0)	51.5	−4.9 (−7.8 to −2.0)	45.3	−4.7 (−7.9 to −1.6)
Q3	**47.5**	**−12.6 (−15.2 to −9.9)**	52.8	1.1 (−1.8 to 4.1)	44.5	−17.0 (−22.7 to −11.3)	48.6	−7.9 (−10.8 to −5.0)	42.9	−7.1 (−10.5 to −3.7)
Q4 (highest)	**42.0**	**−18.1 (−21.1 to −15)**	47.7	−4.0 (−6.0 to −2.1)	37.3	−24.2 (−27.9 to −20.5)	42.2	−14.2 (−17.1 to −11.3)	41.0	−9.0 (−12.8 to −5.2)
**SVI related to racial and ethnic minority residents and English proficiency**										
Q1 (lowest)	**45.3**	**Ref**	—	—	48.7	Ref	46.5	Ref	43.9	Ref
Q2	**47.4**	**2.1 (−1.2 to 5.3)**	—	—	52.9	4.3 (−2.2 to 10.7)	46.2	−0.3 (−5.1 to 4.6)	45.3	1.4 (−1.9 to 4.6)
Q3	**51.6**	**6.3 (2.0 to 10.5)**	61.0	Ref	55.4	6.7 (2.5 to 10.9)	51.5	5.1 (−1.8 to 11.9)	43.4	−0.5 (−4.9 to 3.8)
Q4 (highest)	**56.5**	**11.2 (6.4 to 15.9)**	57.9	−3.2 (−9.9 to 3.5)	59.1	10.4 (4.1 to 16.7)	53.3	6.8 (−0.3 to 14.0)	43.6	−0.4 (−5.7 to 5.0)
**SVI related to housing type and transportation**										
Q1 (lowest)	**53.2**	**Ref**	—	—	55.7	Ref	47.8	Ref	47.2	Ref
Q2	**52.7**	**−0.5 (−3.9 to 2.9)**	54.4	Ref	58.4	2.8 (−2.0 to 7.5)	50.0	2.2 (−2.8 to 7.2)	44.5	−2.7 (−5.5 to 0.2)
Q3	**53.4**	**0.2 (−3.4 to 3.9)**	54.9	0.4 (−5.8 to 6.7)	58.2	2.5 (−1.8 to 6.9)	52.6	4.8 (0.0 to 9.6)	43.5	−3.7 (−6.2 to −1.1)
Q4 (highest)	**55.1**	**1.9 (−2.2 to 5.9)**	60.2	5.8 (−1.0 to 12.6)	56.1	0.4 (−7.4 to 8.2)	51.6	3.8 (−1.2 to 8.8)	43.0	−4.2 (−7.7 to −0.7)

**Abbreviations:** CI = confidence interval; Ref = referent group; SVI = social vulnerability index; VC = vaccination coverage.

* Scores for all SVI measures represent percentile ranks by county ranging from 0–1 with higher scores indicating higher vulnerability. Scores were categorized into quartiles based on distribution among all 3,142 U.S. counties and then applied to the 3,129 assessed counties.

^†^ Vaccination coverage (≥1 dose) was calculated by summing the number of vaccinated adults in each SVI category and dividing by the total adult population in the specified SVI category. 95% CIs for the vaccination coverage differences were calculated using generalized estimating equation models with robust standard errors to account for state variability.

^§^ Urbanicity categories were based on the 2013 National Center for Health Statistics urban-rural classification scheme (https://www.cdc.gov/nchs/data/series/sr_02/sr02_166.pdf). Categories were collapsed into large metropolitan, large fringe metropolitan, medium and small metropolitan, and nonmetropolitan (micropolitan and noncore) counties.

^¶^ California counties with populations <20,000 (n = 8) and all Hawaii counties (n = 5) were excluded because of lack of available county-level vaccination data.

** Results were suppressed for SVI and urbanicity categories with four or fewer counties; reference group was the lowest vulnerability quartile with more than four counties.

Individual components of SVI themes related to socioeconomic status and housing composition and disability highlighted factors contributing to disparities. Vaccination coverage was lower among adults living in counties with per capita income less than the median (42.7%) compared with those in counties at or above the median (56.7%) and other social determinants of poor health, including poverty and less education, especially in large fringe metropolitan and nonmetropolitan counties (Table 2). Vaccination coverage was also lower among adults living in counties where the percentages of children, persons with disabilities, or single-parent households were at or above the median (51.3%, 43.9%, and 51.5%, respectively) compared with those in counties where the percentages of these groups were below the median (56.8%, 56.3%, and 58.0%, respectively), especially in large fringe metropolitan counties. Although coverage did not vary by the SVI theme related to housing type and transportation, one component of this theme suggested disparities in coverage. Specifically, vaccination coverage was lower in counties where the percentage of mobile homes was at or above the median (42.1%) compared with those where this percentage was below the median (58.8%).

**TABLE 2:** Associations between individual components of the social vulnerability index* and vaccination coverage^†^ among U.S. adults, overall and by urbanicity^§^ (N = 3,129 counties^¶^) — United States, December 14, 2020–May 1, 2021

SVI indicator	All counties		Large central metropolitan		Large fringe metropolitan		Medium and small metropolitan		Nonmetropolitan	
	(N = 3,129)		(n = 68)		(n = 368)		(n = 727)		(n = 1,966)	
	VC estimate	VC differences (95% CI)	VC estimate	VC differences (95% CI)	VC estimate	VC differences (95% CI)	VC estimate	VC differences (95% CI)	VC estimate	VC differences (95% CI)
**SVI related to socioeconomic status**										
**Percentage of persons living below poverty (median = 14.7%)**										
Below median	**57.4**	**Ref**	63.9	Ref	58.5	Ref	54.4	Ref	49.1	Ref
At or above median	**49.8**	**−7.7 (−10.0 to −5.3)**	54.8	−9.1 (−15.3 to −2.9)	45.9	−12.7 (−15.6 to −9.7)	48.6	−5.8 (−8.9 to −2.7)	40.8	−8.3 (−11.2 to −5.5)
**Percentage of persons unemployed (median = 5.4%)**										
Below median	**56.6**	**Ref**	61.4	Ref	60.0	Ref	53.3	Ref	47.0	Ref
At or above median	**51.9**	**−4.7 (−6.7 to −2.7)**	56.5	−4.9 (−8.6 to −1.1)	52.9	−7.1 (−9.8 to −4.4)	50.4	−3.0 (−5.9 to 0.0)	42.0	−5.0 (−7.8 to −2.3)
**Income per capita (median = $26,245)**										
At or above median	**56.7**	**Ref**	—**	—	58.6	Ref	53.9	Ref	50.9	Ref
Below median	**42.7**	**−14 (−16.5 to −11.5)**	—	—	41.6	−16.9 (−20.7 to −13.2)	45.1	−8.8 (−12.4 to −5.2)	40.2	−10.7 (−13.3 to −8.2)
**Percentage of persons aged ≥25 years with no high school diploma (median = 12.1%)**										
Below median	**56.5**	**Ref**	60.1	Ref	59.4	Ref	53.7	Ref	49.9	Ref
At or above median	**50.4**	**−6.2 (−9.2 to −3.1)**	56.8	−3.3 (−7.5 to 1.0)	47.6	−11.8 (−15.4 to −8.3)	47.1	−6.5 (−10.8 to −2.3)	39.8	−10.2 (−12.7 to −7.6)
**SVI related to household composition and disability**										
**Percentage of persons aged ≥65 years (median = 18%)**										
Below median	**54.9**	**Ref**	57.9	Ref	57.5	Ref	51.7	Ref	42.8	Ref
At or above median	**49.4**	**−5.5 (−8.1 to −3.0)**	61.0	3.1 (−5.3 to 11.5)	54.9	−2.5 (−6.7 to 1.7)	50.6	−1.1 (−4.1 to 1.9)	45.2	2.4 (−0.1 to 4.9)
**Percentage of persons aged <18 years (median = 22.3%)**										
Below median	**56.8**	**Ref**	63.1	Ref	60.7	Ref	53.6	Ref	45.8	Ref
At or above median	**51.3**	**−5.5 (−7.8 to −3.3)**	53.3	−9.7 (−11.4 to −8.1)	54.8	−6.0 (−11.0 to −0.9)	49.6	−4 (−6.4 to −1.7)	42.1	−3.7 (−6.2 to −1.3)
**Percentage of persons living with a disability (median = 15.4%)**										
Below median	**56.3**	**Ref**	58.2	Ref	58.1	Ref	53.8	Ref	47.7	Ref
At or above median	**43.9**	**−12.4 (−15.1 to −9.7)**	51.7	−6.5 (−12.3 to −0.8)	43.7	−14.4 (−19.2 to −9.6)	44.7	−9.1 (−12.3 to −5.8)	42.0	−5.7 (−8.4 to −3.0)
**Percentage of households with single parents and children (median = 8.1%)**										
Below median	**58.0**	**Ref**	65.3	Ref	62.4	Ref	54.7	Ref	45.5	Ref
At or above median	**51.5**	**−6.5 (−8.3 to −4.6)**	55.7	−9.6 (−11.6 to −7.6)	51.5	−10.9 (−13.8 to −8.0)	49.9	−4.8 (−7.3 to −2.3)	43.0	−2.4 (−4.7 to −0.1)
**SVI related to racial and ethnic minority residents and English proficiency**										
**Percentage of racial and ethnic minority residents (median = 16.1%)**										
Below median	**48.5**	**Ref**	—	—	53.7	Ref	49.2	Ref	45.1	Ref
At or above median	**55.1**	**6.6 (3.2 to 10.1)**	—	—	57.9	4.2 (0.5 to 7.9)	52.1	2.9 (−1.1 to 6.9)	42.9	−2.2 (−5.9 to 1.4)
**Percentage of persons who speak English less than well (median = 0.7%)**										
Below median	**45.8**	**Ref**	—	—	50.3	Ref	45.7	Ref	43.9	Ref
At or above median	**55.2**	**9.5 (6.4 to 12.5)**	—	—	58.1	7.7 (4.4 to 11.1)	52.5	6.8 (3.4 to 10.2)	44.3	0.4 (−2.5 to 3.3)
**SVI related to housing type and transportation**										
**Percentage of housing structures with ≥10 units (median = 2.9%)**										
Below median	**40.9**	**Ref**	—	—	40.7	Ref	42.3	Ref	40.4	Ref
At or above median	**55.4**	**14.5 (11.9 to 17.1)**	—	—	58.3	17.7 (14.8 to 20.6)	52.2	9.9 (5.9 to 13.9)	47.4	7.0 (5.0 to 8.9)
**Percentage of housing units that are mobile home units (median = 10.9%)**										
Below median	**56.4**	**Ref**	—	—	58.8	Ref	53.6	Ref	49.7	Ref
At or above median	**42.0**	**−14.4 (−17.2 to −11.5)**	—	—	42.1	−16.7 (−20.6 to −12.8)	44.3	−9.3 (−12.5 to −6.1)	40.0	−9.7 (−12.5 to −6.8)
**Percentage of households with more persons than rooms (median = 1.9%)**										
Below median	**53.4**	**Ref**	57.6	Ref	58.4	Ref	51.9	Ref	45.8	Ref
At or above median	**54.1**	**0.7 (−2.7 to 4.2)**	58.0	0.4 (−5.4 to 6.2)	56.3	−2.1 (−5.6 to 1.4)	51.2	−0.7 (−3.9 to 2.4)	42.7	−3.1 (−5.8 to −0.5)
**Percentage of households with no vehicle access (median = 5.7%)**										
Below median	**53.7**	**Ref**	63.9	Ref	55.9	Ref	50.3	Ref	44.6	Ref
At or above median	**54.0**	**0.3 (−3.0 to 3.5)**	56.7	−7.2 (−12.5 to −1.9)	59.3	3.4 (0.0 to 6.8)	52.1	1.8 (−2.2 to 5.8)	43.8	−0.8 (−3.4 to 1.7)
**Percentage of persons living in institutionalized group quarters (median = 2.0%)**										
Below median	**53.5**	**Ref**	56.0	Ref	56.3	Ref	50.5	Ref	43.5	Ref
At or above median	**54.3**	**0.9 (−1.2 to 2.9)**	61.4	5.4 (0.4 to 10.5)	58.9	2.6 (−2.6 to 7.8)	52.0	1.6 (−1.5 to 4.7)	44.6	1.1 (−0.9 to 3.0)

**Abbreviation:** CI = confidence interval; Ref = referent group; SVI = social vulnerability index; VC = vaccination coverage.

* Scores for all SVI measures represent percentile ranks by county ranging from 0–1 with higher scores indicating higher vulnerability. Scores were categorized into quartiles based on distribution among all 3,142 U.S. counties and then applied to the 3,129 assessed counties.

^†^ Vaccination coverage (≥1 dose) was calculated by summing the number of vaccinated adults in each SVI category and dividing by the total adult population in the specified SVI category. 95% CIs for the vaccination coverage differences were calculated using generalized estimating equation models with robust standard errors to account for state variability.

^§^ Urbanicity categories were based on the 2013 National Center for Health Statistics urban-rural classification scheme (https://www.cdc.gov/nchs/data/series/sr_02/sr02_166.pdf). Categories were collapsed into large metropolitan, large fringe metropolitan, medium and small metropolitan, and nonmetropolitan (micropolitan and noncore) counties.

^¶^ California counties with populations <20,000 (n = 8) and all Hawaii counties (n = 5) were excluded because of lack of available county-level vaccination data.

** Results were suppressed for SVI and urbanicity categories with four or fewer counties; reference group was the lowest vulnerability quartile with more than four counties.

## Discussion

Counties with higher SVIs have been disproportionately affected by the COVID-19 pandemic (*4*); therefore, ensuring equitable access to COVID-19 vaccination is a priority for the U.S. COVID-19 vaccination program (*5*). In addition, disparities in vaccination coverage by SVI have increased over time, especially in large fringe metropolitan and nonmetropolitan counties. Disparities were associated with county-level differences in socioeconomic status and household composition and disability. Although disparities were not associated with county-level differences related to racial and ethnic minority residents and housing types, individual SVI components suggested disparities among adults living in counties with particular housing characteristics (e.g., lower coverage in counties with higher percentages of mobile homes). These results underscore the importance of timely strategies to ensure that all communities can equitably benefit from COVID-19 vaccination.

Although differences in coverage by SVI were observed in counties of all urbanicity levels, large fringe metropolitan and nonmetropolitan counties were most affected. Persons living in these counties might experience unique challenges in accessing vaccination. For example, residents of large fringe metropolitan counties might face socioeconomic challenges, including substantial barriers to accessing health care services (*6*,*7*). COVID-19 vaccination coverage has been lower in rural than in urban areas, and persons in rural areas are more likely to travel outside their county of residence for vaccination (*8*). Efforts to improve vaccination coverage could focus on areas that are more vulnerable with respect to socioeconomics and household composition, while tailoring interventions by urbanicity.

Focused efforts to increase access to vaccination could help ensure high and equitable vaccination coverage. Opportunities to increase access by enrolling providers who are known and trusted in the community and partnering with community- and faith-based organizations to organize pop-up clinics*** should be considered. Mobile and walk-in vaccination clinics with flexible evening and weekend hours could also increase access in such communities.^†††^ Home visits, although resource-intensive, have proven effective at increasing non–COVID-19 vaccination coverage among adults (*9*). Establishing COVID-19 vaccination clinics near child care facilities and schools, with hours communicated to parents through school channels, could increase vaccination coverage among adults in single-parent households. Vaccination locations should be accessible to persons with disabilities and offer special hours for persons who require extra assistance.

Because U.S. adults with less education and income and without health insurance were more likely to report vaccine hesitancy before the start of the COVID-19 vaccination program (*10*), strategies to improve vaccination coverage in counties with high SVI should also address vaccine confidence. This might include involving trusted messengers from the community who can communicate vaccine concerns, such as vaccine side effects or risk, and promote the benefit of immunization using local communication platforms.^§§§^ For example, expanded public health messaging campaigns in a variety of accessible formats could raise awareness that the vaccine is free, safe, effective, and necessary to decrease COVID-19 incidence in local communities.

The findings in this report are subject to at least four limitations. First, because SVI and vaccination coverage might have varied within counties, additional analyses could account for a finer geographic scale. Second, disparities in coverage by SVI might have differed if vaccination series completion had been assessed. Third, sparse data for certain SVI and urbanicity categories limited interpretation of results. Finally, the findings provide only a national picture of COVID-19 vaccination coverage by SVI, and state-specific patterns should be explored to direct efforts to local areas.

COVID-19 vaccination coverage disparities by SVI have persisted and increased over time, even as vaccination eligibility and access have expanded. Disparities are associated with socioeconomic status and household composition and disability, particularly in large fringe metropolitan areas. Ensuring equitable COVID-19 vaccine access will require focused efforts on increasing coverage in counties with high SVI and tailoring efforts to local population needs. Efforts could include walk-in vaccination clinics and public health messaging about the importance of getting vaccinated.

**Summary**

**What is already known about this topic?**

Counties with higher levels of social vulnerability have been disproportionately affected by COVID-19.

**What is added by this report?**

Disparities in county-level vaccination coverage by social vulnerability have increased as vaccine eligibility has expanded, especially in large fringe metropolitan (areas surrounding large cities, e.g., suburban) and nonmetropolitan counties. By May 1, 2021, vaccination coverage among adults was lower among those living in counties with lower socioeconomic status and with higher percentages of households with children, single parents, and persons with disabilities.

**What are the implications for public health practice?**

Outreach efforts, including expanding public health messaging tailored to local populations and increasing vaccination access, could help increase vaccination coverage in counties with high social vulnerability.

## Footnotes

* These authors contributed equally to this report.

† Vaccination coverage was calculated by summing the number of vaccinated adults in each SVI category and dividing by the total adult population in the specified SVI category. Population denominators were obtained from the U.S. Census Bureau.

§ Urbanicity was defined on the basis of the 2013 National Center for Health Statistics urban-rural classification scheme. For this analysis, categories included large central metropolitan counties, large fringe metropolitan counties, medium and small metropolitan counties, and nonmetropolitan counties. Large central metropolitan counties are counties in metropolitan statistical areas (MSAs) with ≥1 million population; large fringe metropolitan counties are counties in MSAs with ≥1 million population that did not qualify as large central metropolitan counties; medium metropolitan counties are counties in MSAs with populations of 250,000–999,999; small metropolitan counties are counties in MSAs with populations <250,000; nonmetropolitan counties are all micropolitan and noncore counties. https://www.cdc.gov/nchs/data_access/urban_rural.htm

¶ Entities including jurisdictions, pharmacies, and federal agencies reported vaccinations to CDC. A cloud-hosted data repository received, deduplicated, and deidentified vaccination data. https://www.cdc.gov/coronavirus/2019-ncov/vaccines/distributing/about-vaccine-data.html

** The 15 population-based social factors incorporated into the SVI measures included 1) percentage of persons with incomes below poverty threshold, 2) percentage of civilian population (aged ≥16 years) that is unemployed, 3) per capita income, 4) percentage of persons aged ≥25 years with no high school diploma, 5) percentage of persons aged ≥65 years, 6) percentage of persons aged ≤17 years, 7) percentage of civilian noninstitutionalized population aged >5 years with a disability, 8) percentage of single-parent households with children aged <18 years, 9) percentage of persons who are racial/ethnic minorities (i.e., all persons except those who are non-Hispanic White), 10) percentage of persons aged ≥5 years who speak English “less than well,” 11) percentage of housing in structures with ≥10 units (multiunit housing), 12) percentage of housing structures that are mobile homes, 13) percentage of households with more persons than rooms (crowding), 14) percentage of households with no vehicle available, and 15) percentage of persons living in institutionalized group quarters. Estimates were created using 2014–2018 (5-year) data from the American Community Survey (https://www.atsdr.cdc.gov/placeandhealth/svi/documentation/pdf/SVI2018Documentation-H.pdf). The 15 indicators are categorized into four themes: 1) socioeconomic status (indicators 1–4), 2) household composition and disability (indicators 5–8), 3) racial/ethnic minority status and language (indicators 9 and 10), and 4) housing type and transportation (indicators 11–15). Overall SVI includes all 15 indicators as a composite measure. https://www.atsdr.cdc.gov/placeandhealth/svi/fact_sheet/fact_sheet.html

†† 95% CIs for the vaccination coverage differences used robust standard errors to account for state variability.

§§ Periods used in the Supplementary Table were December 14, 2020–January 23, 2021; January 24–March 20, 2021; and March 21–May 1, 2021.

¶¶ 45 C.F.R. part 46, 21 C.F.R. part 56; 42 U.S.C. Sect. 241(d); 5 U.S.C. Sect. 552a; 44 U.S.C. Sect. 3501 et seq.

*** Pop-up clinics can operate from any publicly accessible space, and be staffed by physicians, nurses, and volunteers. https://www.cdc.gov/vaccines/covid-19/downloads/Key-Op-Considerations-COVID-Mass-Vax.pdf

†††
https://www.cdc.gov/vaccines/covid-19/planning/mobile.html

§§§
https://www.cdc.gov/vaccines/covid-19/vaccinate-with-confidence.html
